# Promising Anti-Wrinkle Applications of Aromatic Extracts of *Hedychium coronarium* J. Koenig via Antioxidation and Collagenase Inhibition

**DOI:** 10.3390/ph16121738

**Published:** 2023-12-17

**Authors:** Pattiya Tammasorn, Wannaree Charoensup, Anurak Bunrod, Watchara Kanjanakawinkul, Wantida Chaiyana

**Affiliations:** 1 Department of Pharmaceutical Sciences, Faculty of Pharmacy, Chiang Mai University, Chiang Mai 50200, Thailand; pattiya_tammasorn@cmu.ac.th (P.T.); wannaree.charoensup@cmu.ac.th (W.C.); 2 Chulabhorn Royal Pharmaceutical Manufacturing Facilities by Chulabhorn Royal Academy, Chon Buri 20180, Thailand; anurak.bun@cra.ac.th (A.B.); watchara.kan@cra.ac.th (W.K.)

**Keywords:** *Hedychium coronarium*, concrete, absolute, essential oil, aroma, terpenes, antioxidant, anti-skin wrinkle, cosmetics

## Abstract

This study aimed to extract aromatic compounds from the rhizomes, leaf sheaths, and leaves of *Hedychium coronarium* and investigate their chemical compositions, cosmetic/cosmeceutical activities, and irritation potency. The chemical compositions were investigated via gas chromatography–mass spectrometry. The antioxidant activities were evaluated via spectrophotometry. The anti-skin wrinkle properties were investigated via collagenase, elastase, and hyaluronidase inhibition. The irritation potency was observed via a hen’s egg–chorioallantoic membrane test. Eucalyptol was detected as a major component in the rhizomes and leaf sheaths, while β-caryophyllene was predominant in the leaves. The absolutes from the rhizomes were the strongest antioxidants, with ABTS scavenging properties similar to *L*-ascorbic acid. Interestingly, the equivalent concentration (EC_1_) of the absolute from the rhizome was 0.82 ± 0.01 µg FeSO_4_/g extract, which was significantly more potent than *L*-ascorbic acid (0.43 ± 0.03 µg FeSO_4_/g extract). The rhizome-derived absolute was the most effective against collagenase, while the concretes from the rhizomes and leaf sheaths showed promising anti-hyaluronidase activity with inhibitions of 90.5 ± 1.6% and 87.4 ± 5.1%, respectively. The irritability of the aromatic extracts was not different from that of the vehicle control, proving their safety. Therefore, the *Hedychium coronarium* rhizome-derived absolute was an attractive and potent antioxidant with anti-collagenase activities, indicating its potential for use in anti-aging formulations.

## 1. Introduction

The Zingiberaceae family, known for its medicinal benefits, can be found in various tropical regions, with a significant presence in Southeast Asia [1]. The plants in the Zingiberaceae family are often rhizomatous annual or perennial herbs with considerable economic potential that are utilized as vegetables, spices, sauces, flavorings, medications, and colors [2,3]. One of its numerous applications, which could strongly enhance the worth of the plants, is the production of fragrances. *Hedychium coronarium* J. Koenig, commonly known as butterfly ginger lily, is a plant belonging to the Zingiberaceae family that is cultivated as a garden plant and for cutting flowers, both for decoration and the perfume industry [4,5]. The essential oil from the *H. coronarium* flower has been used in high-quality fragrances [6,7,8]. Aside from fragrances, the flower’s essential oil has long been used in traditional medicine to treat inflammation, skin conditions, headaches, and severe rheumatic pain [9]. In addition, the essential oils were also used as fragrances in various cosmetic formulations, including cleansing products and lotions [6,7,8,9,10].

Regarding the utilization of *H. coronarium* flowers in the perfume industry, the remaining parts of the plants have been disposed of every year. Rhizomes, leaf sheaths, leaves, etc., have been discarded as agricultural waste and have no value. However, the rhizomes of *H. coronarium* have been reported to contain more plentiful amounts of volatile oils compared to the other parts of the plant [11]. Eucalyptol, *β*-pinene, *α*-pinene, and *α*-terpineol, which have been found as the major constituents in rhizome essential oils, are widely acknowledged to have a variety of biological activities, including antibacterial, antioxidant, larvicidal, phytotoxic, anthelmintic, and anti-inflammatory activities [6,7,8]. On the other hand, there have been some studies indicating that the extracts from the rhizomes and leaves of *H. coronarium* had excellent antioxidant properties [12]. Consequently, *H. coronarium* rhizomes, leaf sheaths, and leaves, which have been dumped as waste from the perfume industry, may be employed as active ingredients in the cosmetic and cosmeceutical areas.

The plants in the Zingiberaceae have been widely known to contain volatile components, not only in the flowers but also in other parts [13]. Therefore, the aromatic extracts from *H. coronarium* are of interest. Essential oils, which are volatile liquids typically transparent and seldom colored, are the most commonly used among the aromatic extracts from plants. Valuable aromatic materials, apart from essential oils, include the concrete and absolute. The concrete is a semisolid mass produced by the solvent extraction of plant material in the fragrance industry. Various nonpolar solvents have been used to produce concrete, such as hexane, benzene, and toluene, due to their ability to dissolve the waxy and fragrant plant compounds [14]. The solvent is finally removed to leave a solid or semisolid substance known as concrete. Meanwhile, the absolute is obtained by extracting the concrete using ethanol [14]. The ethanolic extract is then cooled to allow the waxes to solidify. The solidified wax is filtered out, and the solvent is evaporated under vacuum conditions, leaving behind the absolute, which is characterized by its intense aromatic properties and high concentration.

As a large amount of the *H. coronarium* was left over from the perfume industry, the present study aimed to utilize these agricultural waste products as a source of biologically active aromatic compounds for the cosmetic/cosmeceutical industry. The concretes, absolutes, and essential oils from the rhizomes, leaf sheaths, and leaves of *H. coronarium* were investigated for their chemical compositions, biological activities related to the cosmetic/cosmeceutical applications, and irritation potency. 

## 2. Results and Discussion

### 2.1. Plant Identifications

**Figure 1:**
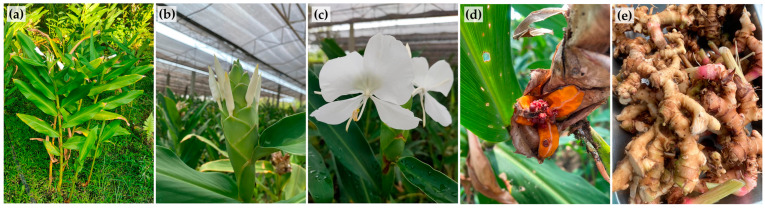
*H. Coronarium* growing in the field (**a**), inflorescence with flower buds (**b**), flowers (**c**), split ripened capsules with seeds (**d**), and rhizomes (**e**).

*H. Coronarium* belongs to the Zingiberaceae family. The Hedychium genus contains about 50 species distributed in tropical Asia and Madagascar [4]. *H. Coronarium* has the greatest accessibility and prevalence, attributed to its robust characteristics and fragrant blossoms [4]. It is characterized by the bright green stem and its bright green leaves, which are distichous lanceolate (Figure 1a) [4]. The flowers are typically arranged in terminal spikes, with more than one flower on each spike, typically each having 2–4 flowers (Figure 1b) [15]. The flowers are white, broadly orbicular, and sweetly scented (Figure 1c) [16]. The yellowish fleshy portion of the oval fruits with 3-valved capsules, which contain a huge number of seeds covered with red-colored aril, breaks apart when ripened (Figure 1d) [15,17]. The rhizomes are fleshy, branched, and knotty, with many nodes that extend horizontally beneath the soil surface (Figure 1e) [15].

**Figure 2:**
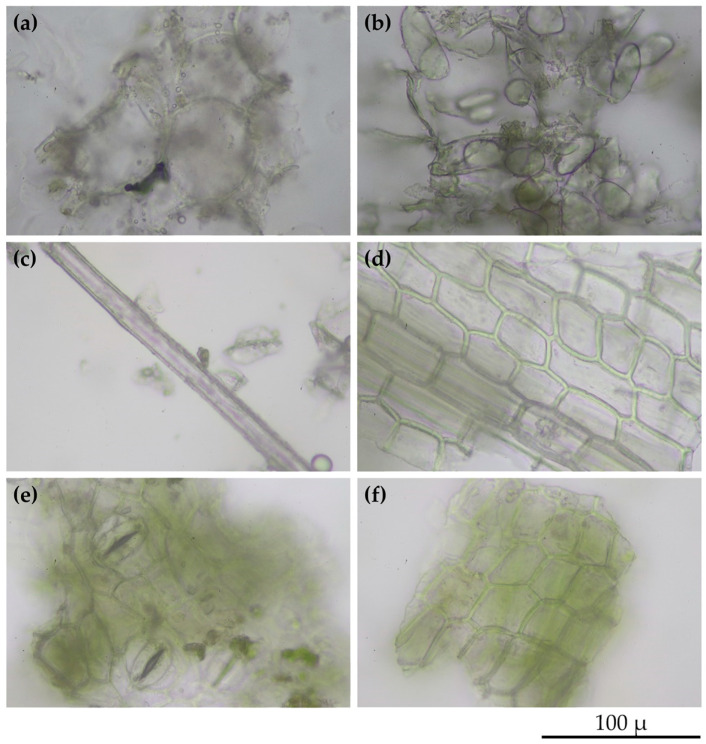
Microscope images of *H. Coronarium* rhizome powder presenting parenchyma with intercellular space containing oil droplets (**a**) and parenchyma containing starch granules (**b**); *H. Coronarium* leaf sheath powder presenting fiber and oil droplets (**c**) and polygonal epidermis (**d**); *H. Coronarium* leaf powder presenting lower epidermis showing stomata (**e**) and upper with palisade mesophyll in sectional view (**f**).

Since *H. coronarium* has been well known for its significance in flower fragrances, it is currently used in the perfume industry. Aside from the flowers, the other parts also contain aromatic compounds. Essential oils from the rhizome, stem, and leaf of *H. coronarium* have been reported to be successfully extracted and contain a variety of volatile constituents [18]. Hence, various parts of *H. coronarium*, including the rhizomes, leaf sheaths, and leaves, were used in the present study. The microscope images of dried powder from various parts of *H. coronarium* are shown in Figure 2. Oil droplets were detected in the powder of the rhizome and leaf sheath, as shown in Figure 2a,c, respectively. On the other hand, starch granules were detected in the rhizome part. The polygonal epidermis was detected in both the leaf sheaths and leaves, while stomata was only found in the leaf part.

### 2.2. Aromatic Extracts from H. coronarium 

**Figure 3:**
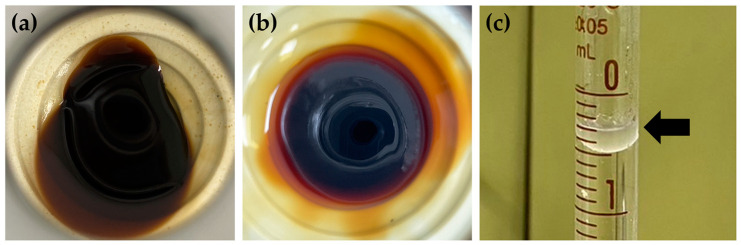
Physical appearance of concrete (**a**), absolute (**b**), and the essential oil, indicated by the black arrow (**c**), of *H. coronarium* rhizome.

**Figure 4:**
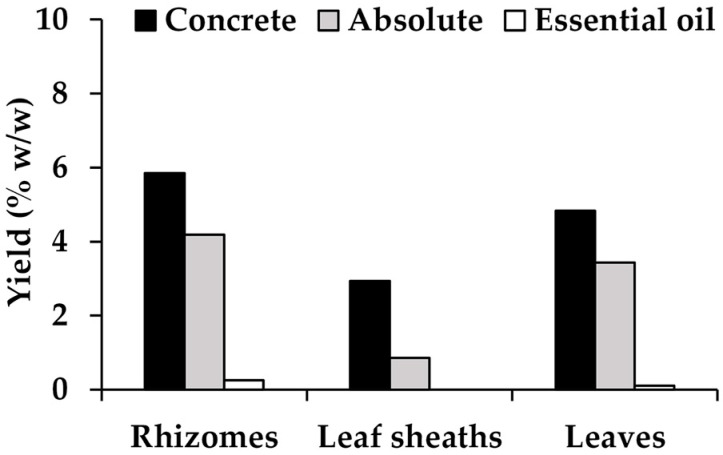
The yields of *H. coronarium* aromatic extracts reported as percentages based on the dried plant materials.

Various aromatic extracts from the *H. coronarium* rhizomes, leaf sheaths, and leaves were prepared in terms of the concrete, absolute, and essential oils, as shown in Figure 3. As concretes are extracted from aromatic plant materials using nonpolar volatile solvents, they are solid wax-like substances that not only contain volatiles, such as terpenes and sesquiterpenes, but also fatty acids and their methyl esters, paraffins, and other high-molecular-weight chemicals [19]. The concretes from *H. coronarium* were found to be dark brown semisolid masses with high viscosity, and their odor varied depending on the plant part. On the other hand, absolutes are wax-free aromatic substances prepared by removing the wax from the concrete via ethanol extraction [19]. The absolutes from *H. coronarium* were also found to be a dark brown color as semisolid masses but with less viscosity compared to the concrete. A unique scent was found from each absolute, depending on the plant parts used. In contrast to the concrete and absolutes, the essential oils were from hydrodistillation, and the oils were transparent liquids with a very light-yellow color. The essential oil yields were the lowest compared to the concrete and absolutes. No essential oil was obtained from the leaf sheaths of *H. coronarium* based on the hydrodistillation of 100 g of dried plant materials. The yields of the aromatic extracts from *H. coronarium* are shown in Figure 4. The concrete yields were the greatest among all the aromatic extracts from *H. coronarium*, followed by the absolutes and essential oils. The rhizomes had the largest concrete production (5.85% *w*/*w*), followed by the leaves (4.83% *w*/*w*) and leaf sheaths (2.94% *w*/*w*). The yields of the absolutes followed the same pattern as the concretes. The rhizomes had the largest absolute yield (4.19% *w*/*w*), followed by the leaves (3.43% *w*/*w*) and leaf sheaths (0.86% *w*/*w*). The absolute yields from the rhizomes and leaves were approximately 71% *w/w* of their concrete yields. In contrast, the absolute leaf sheath yield was only 29.25% *w*/*w* of the concrete. The essential oil yields were the lowest. Those from the rhizomes and leaves yielded only 0.25 and 0.10% *w*/*w*, respectively.

### 2.3. Chemical Compositions of H. coronarium

The chemical compositions of the aromatic extracts from the *H. coronarium* rhizomes, leaf sheaths, and leaves are shown in Table 1. Different chemical profiles were observed in different parts of the plant. Eucalyptol was found as the major constituent in all the aromatic extracts from the rhizome part. The absolute contained the highest content of eucalyptol (72.9 ± 1.9%), followed by the concrete (67.8 ± 1.0%) and essential oil (53.6 ± 0.5%), respectively. The results were in line with the previous study, which reported that eucalyptol was the major component in essential oils from *H. coronarium* rhizomes [4]. However, the eugenol content in the previous study was found to be lower, ranging from 11.48 to 40.59% [4]. However, the essential oils of *H. coronarium* populations could be grouped into four different chemotypes, including eucalyptol, linalool, coronarin-E, or β-pinene [4,20]. In the case of the present study, eucalyptol was the predominant compound, with β-pinene present in relatively low concentrations, linalool was detected in trace amounts, and coronarin-E was not identified. Similar to the rhizome part, eucalyptol was also found predominantly in the leaf sheaths but in a lower content. In contrast, β-caryophyllene was found to be the major constituent in the aromatic extracts from the *H. coronarium* leaves, especially in the absolute. The results were in line with the previous study, which reported that β-caryophyllene was the most predominant in the leaves of *H. coronarium* [21]. However, β-pinene was found to be the most abundant compound in the essential oil from the *H. coronarium* leaves. It was also noted that different types of aromatic extracts also contained different chemical profiles. It was obviously observed that some low-molecular-weight aromatic compounds were absent in the absolute. The most plausible reason is that these chemicals evaporated during the process of evaporation to remove ethanol following the wax removal from the concrete. Although the temperature of the water bath equipped with the rotary evaporator was set low, around 40 °C, the vacuum conditions resulted in high pressure within the system, thereby lowering the boiling point, which refers to the temperature at which a liquid transforms into a gas of compounds. For instance, ethanol, which has a boiling point of 78.37 °C [22], could evaporate at this condition. Consequently, volatile compounds with a low molecular weight, typically associated with low boiling points, could have consequently evaporated [23]. Therefore, the absolutes were rich in the larger molecules of the aromatic compounds. Eucalyptol reached an impressive concentration of 72.9 ± 1.9% in the *H. coronarium* rhizome absolute, while the *H. coronarium* leaf absolute contained a remarkable 84.2 ± 0.7% of β-caryophyllene. In contrast, a higher concentration of a low-molecular-weight aromatic compound was found in the essential oil, especially β-pinene. The reason could be attributed to the nature of the hydrodistillation extraction process, which facilitated the easy volatility and effective extraction of molecules with a low molecular weight. The chemical profile of these aromatic compounds was strongly correlated with the odor of the extract, with the essential oil producing the most potent fragrance.

### 2.4. Antioxidant Activities of H. coronarium Aromatic Extracts

**Figure 5:**
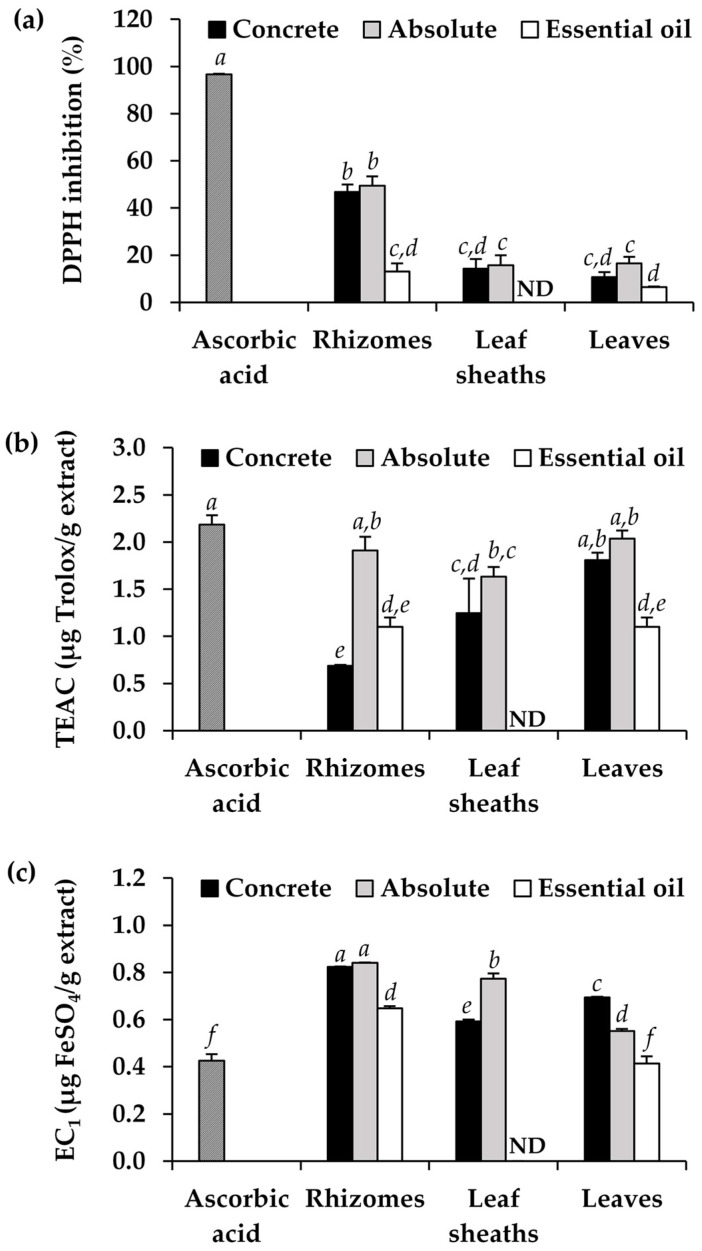
DPPH inhibition (**a**), Trolox equivalent antioxidant capacity (TEAC) (**b**), and equivalent concentration (EC_1_) (**c**) of *L*-ascorbic acid and aromatic extracts from *H. coronarium*. The letters *a*, *b*, *c*, *d*, *e*, and *f* denote significant differences among the antioxidant activities of each sample, analyzed by a one-way ANOVA followed by Tukey’s post-hoc test (*p* < 0.05). ND represents not determined.

The antioxidant activities of the concretes, absolutes, and essential oils from different parts of *H. coronarium* are shown in Figure 5. Three different assays were used to evaluate the antioxidant properties of the aromatic extracts from *H. coronarium*. The 2,2-diphenyl-1-picrylhydrazyl (DPPH) and 2,2′-azino-bis-3-ethylbenzothiazoline-6-sulfonic acid (ABTS) assays were used to evaluate the ability of the extracts to eliminate free radicals, which are related to the electron transfer process [24], whereas the ferric-reducing antioxidant power (FRAP) assay was used to demonstrate the extracts’ capacity to transform the ferric ion (Fe^3+^) to ferrous ion (Fe^2+^) [25]. According to the findings, the *H. coronarium* aromatic extracts showed promising antioxidant properties through their ability to scavenge ABTS radicals and reduce ferric, but they were only moderately effective at scavenging DPPH radicals. The results aligned with a previous study that compared the radical scavenging activities of essential oils from *H. coronarium* rhizomes and revealed that the oils exhibited a higher potency, indicated by lower IC_50_ values, when tested with the ABTS assay in comparison to the DPPH assay [4]. Nonetheless, the absolutes were found to have more potential for free radical scavenging for both ABTS and DPPH. The absolutes from the rhizomes and leaves of *H. coronarium* exhibited comparable ABTS scavenging properties to *L*-ascorbic acid (2.2 ± 0.1 µg 6-hydroxy-2,5,7,8-tetramethylchroman-2-carboxylic acid (Trolox)/g extract) with the Trolox equivalent antioxidant capacity (TEAC) values of 1.9 ± 0.1 and 2.0 ± 0.1 µg Trolox/g extract, respectively. On the other hand, most of the *H. coronarium* aromatic compounds exhibited outstanding ferric-reducing antioxidant powers, especially those from the rhizome part. Both the concrete and absolute from the *H. coronarium* rhizome possessed the significantly highest ability to reduce ferric (*p* < 0.05). Interestingly, the ferric-reducing ability of the aromatic extract from the *H. coronarium* rhizome, particularly the concrete and absolute, was about double that of *L*-ascorbic acid. The equivalent concentration (EC_1_) of the concrete and absolute from the *H. coronarium* rhizome were 0.82 ± 0.01 and 0.82 ± 0.01 µg FeSO_4_/g extract, respectively, while the EC_1_ of *L*-ascorbic acid was 0.43 ± 0.03 µg FeSO_4_/g extract. Despite previous research indicating a stronger correlation between eucalyptol and DPPH antioxidant activity over FRAP [26], additional studies have documented an ability to reduce the Fe (II) ions of eucalyptol. Furthermore, it was noted that FRAP showed a positive correlation with flavonoids and 1,8-cineole [27,28]. The molecular moieties, such as −C=O, −OH, or conjugated double bonds, present in monoterpenes, have been proposed to play a role in the chelation process of Fe (II) [29].

The findings from this study highlighted the potent antioxidant properties exhibited by various aromatic extracts derived from *H. coronarium*, notably the absolutes and extracts obtained from the rhizome parts. However, the effectiveness of antioxidants relies on their ability to reduce or eliminate an excess of reactive oxygen species (ROS) within a biological system [30]. It is important to note that ABTS and DPPH radicals, although stable and commercially available, are not naturally present in biological systems [31]. Therefore, a subsequent cellular analysis was recommended to provide detailed insights into the underlying biological mechanisms of these attributes. Several reagents have been used to detect the intracellular reactive oxygen species (ROS) in the cellular antioxidant activity, e.g., 10-acetyl-3,7-dihydroxyphenoxazine (Amplex Red Reagent), 5-(and-6)-carboxy-2′,7′-dihydrodifluorofluorescein diacetate (H2DFFDA), dihydrorhodamine methyl ester (DHR123), 2′,7′-dichlorofluorescin diacetate (DCFH-DA), malondialdehyde (MDA), etc. [30]. Therefore, the promising candidate aromatic extracts derived from *H. coronarium* were suggested for further cellular analysis.

### 2.5. Anti-Skin Wrinkle Activities of H. coronarium Aromatic Extracts

**Figure 6:**
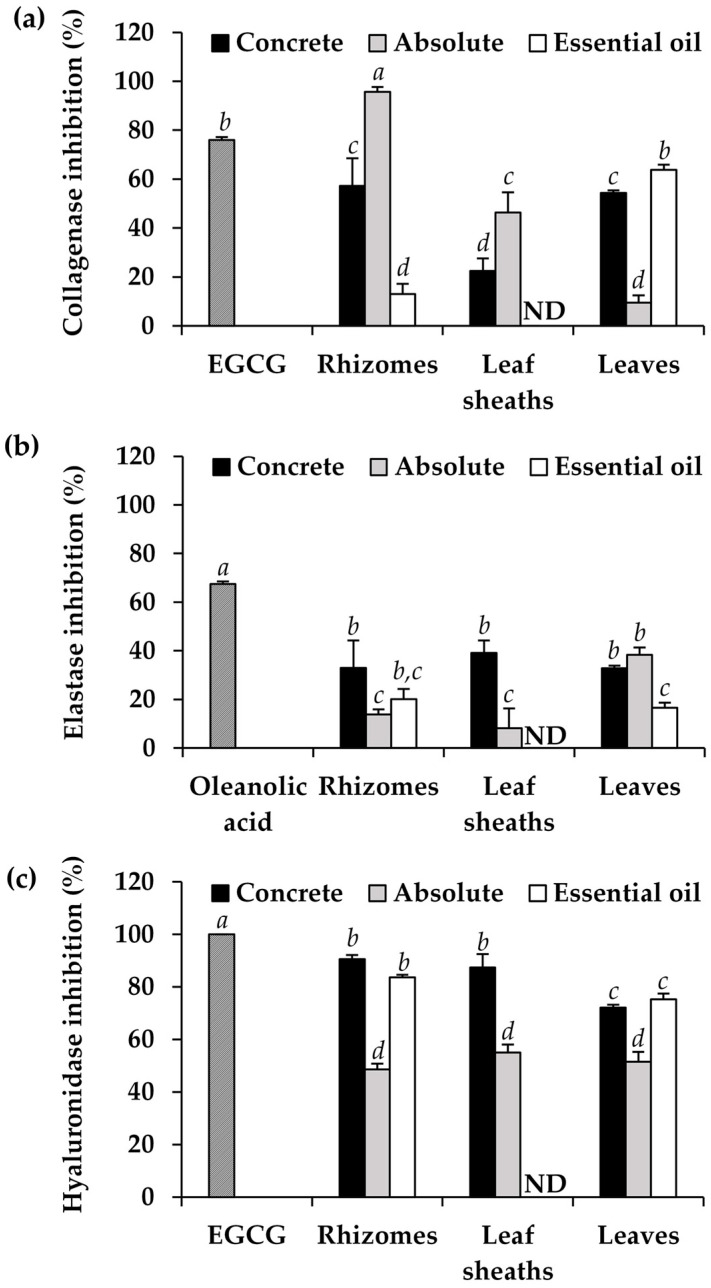
Inhibitory activities against collagenase (**a**), elastase (**b**), and hyaluronidase (**c**) of epigallocatechin gallate (EGCG), oleanolic acid, and aromatic extracts from *H. coronarium*. The letters *a*, *b*, *c*, and *d* denote significant differences among the anti-skin wrinkle activities of each sample, analyzed by a one-way ANOVA followed by Tukey’s post-hoc test (*p* < 0.05). ND represents not determined.

The anti-skin wrinkle activities of the concretes, absolutes, and essential oils from different parts of *H. coronarium* are shown in Figure 6 in terms of the inhibitory activities against collagenase, elastase, and hyaluronidase. The deterioration of the extracellular matrix (ECM), including collagen, elastin, hyaluronic acid, etc., eventually led to sagging skin and wrinkles [32]. The inhibition and retardation of the enzymes responsible for degrading the ECM would be one of the promising approaches for anti-wrinkle. 

The absolute from the *H. coronarium* rhizomes was found to exhibit the most significant potency in inhibiting collagenase (*p* < 0.05). Remarkably, its ability to inhibit collagenase was notably higher than that of EGCG, with inhibitions of 95.7 ± 2.0% and 76.0 ± 1.2%, respectively (*p* < 0.05). The same trend was detected in both the rhizomes and leaf sheaths, showing that the absolute was the most potent, followed by the concrete and essential oil. In contrast, the essential oil from the *H. coronarium* leaves was more potent than the concrete and absolute, respectively. As collagen is the main structural component and the most present ECM of the dermis, it is responsible for strengthening human skin [33]. The absolute from the *H. coronarium* rhizomes, which strongly inhibited collagenase activities, would be an attractive natural aromatic extract proposed for anti-skin wrinkles.

Aside from anti-collagenase activities, the aromatic extracts from *H. coronarium* also inhibited the activities of elastase and hyaluronidase. Elastin fibril is another ECM component that plays a vital role in skin elasticity [34]. Elastin fibril depletion leading to sagging skin is typical for elderly people, primarily due to the loss of skin flexibility [34]. In addition, hyaluronic acid or hyaluronan, a polysaccharide that naturally occurs in the skin, could be readily broken down by hyaluronidase, leading to a loss of skin moisture [35]. The findings from this study demonstrated that the aromatic extracts from *H. coronarium*, particularly concrete, exhibited higher anti-elastase and anti-hyaluronidase activities. The anti-elastase activities of all the *H. coronarium* aromatic extracts were considered moderate. On the other hand, promising anti-hyaluronidase activities were observed. Although the inhibitory effects were not comparable to those of the positive control, the concrete from the *H. coronarium* rhizomes and leaf sheaths exhibited promising anti-hyaluronidase activities, with inhibitions of 90.5 ± 1.6% and 87.4 ± 5.1%, respectively.

Regarding the anti-wrinkle effects concerning the inhibition of collagenase, elastase, and hyaluronidase, the aromatic extracts from *H. coronarium*, particularly the absolute derived from its rhizomes, exhibited a substantial inhibition of collagenase activities. Although the findings from in vitro assays offer intricate insights into the underlying mechanisms of their biological attributes, it is crucial to verify these results through subsequent cellular evaluations. Various cellular assays have been used to assess the potential of natural compounds to exhibit anti-wrinkle properties. The anti-skin aging potency could be assessed by measuring the matrix metalloproteinase-1 (MMP-1) and type I procollagen levels in cultured human dermal fibroblasts using an enzyme-linked immunosorbent assay (ELISA) [36]. The inhibitions against MMP-2 and MMP-9 have been evaluated in mouse embryonic fibroblast (3T3) cells by using sodium dodecyl-sulfate polyacrylamide gel electrophoresis (SDS-PAGE) [37]. Therefore, these cellular assays might be recommended for further investigations of promising aromatic extracts from *H. coronarium*. The findings from the current study would establish a foundation for the selection of candidate aromatic extracts from *H. coronarium* for further comprehensive investigations. 

### 2.6. Irritation Potency of H. coronarium Aromatic Extracts

**Figure 7:**
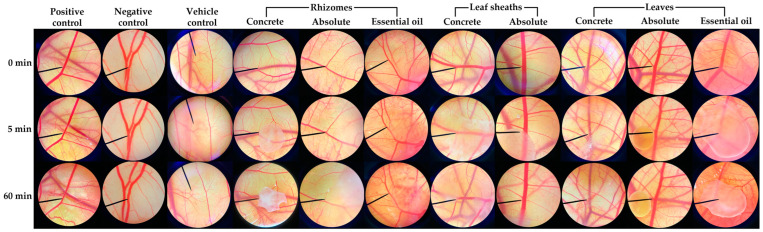
Chorioallantoic membrane after being exposed to a positive control (1% *w*/*v* sodium lauryl sulfate aqueous solution), negative control (0.9% *w*/*v* NaCl aqueous solution), vehicle control (10% *v*/*v* dimethyl sulfoxide in olive oil), and *H. coronarium* aromatic extract at 0, 5, and 60 min.

The irritation potency of the *H. coronarium* aromatic extracts was evaluated after exposure to the chorioallantoic membrane (CAM), as shown in Figure 7, and the irritation levels were classified, as presented in Table 2. Among all the extracts, the concrete from the leaf sheaths of *H. coronarium* induced no irritation. The concrete and essential oil extracted from the rhizomes, as well as the essential oil from the leaves, showed moderate irritation, while the others caused mild irritation. However, no significant difference was observed in the irritation potency among the *H. coronarium* aromatic extracts. Moreover, the irritation of the *H. coronarium* aromatic extracts was not significantly different from that of the vehicle control (10% *v*/*v* dimethyl sulfoxide (DMSO) in olive oil), which was classified as mild irritation with an IS of 4.8 ± 0.0. Therefore, it might be possible that the irritation signs may be attributed to the solvent used rather than the *H. coronarium* extracts themselves. As all the *H. coronarium* aromatic extracts were dissolved in 10% *v*/*v* DMSO in olive oil before being exposed to the CAM, the presence of 10% *v*/*v* DMSO could account for the irritations. The results were in line with the previous study, which reported that more than 1% DMSO was toxic for most mammalian cell types [38]. Even ethanol, which is widely used in topical products, also caused skin irritation or dermatitis [38]. Other irritation tests, such as the human patch test, were suggested to confirm the safety of the *H. coronarium* aromatic extracts.

## 3. Materials and Methods

### 3.1. Plant Materials and Identifications

The whole plant of *H. coronarium* was collected from Sattahip, Chonburi, Thailand, during November 2022. The fresh *H. coronarium* was washed to remove dirt and soil. Each part of *H. coronarium*, including rhizomes, leaf sheaths, and leaves, was manually separated and cut into small pieces. Subsequently, the plant material was dried in a hot-air oven (Memmert, Schwabach, Germany) set at a temperature of 45 °C for 72 h. Then, the dried plant material was ground into fine powder using an electrical blender (Sharp Thai Co., Ltd., Bangkok, Thailand). The dried powder of the *H. coronarium* rhizomes, leaf sheaths, and leaves was kept in a sealed aluminum bag until further experiments. 

To verify the plant species, the entire fresh plant, including flowers, leaves, and rhizomes, was verified by Miss Wannaree Charoensup, a botanist at the Department of Pharmaceutical Science, Faculty of Pharmacy, Chiang Mai University, Thailand. A voucher specimen with the number 0023314 was kept at the herbarium of the Faculty of Pharmacy, Chiang Mai University, Thailand. A small amount of the dried powder of each plant material was mounted in diluted glycerol on the glass slide and characterized under a Nikon ECLIPSE E200 Microscope (Nikon Solutions Co., Ltd., Konan, Japan) connected with a Canon EOS750D camera (Canon Inc., Tochigi, Japan), and the photos were taken with a magnification of 40×. 

### 3.2. Chemical Materials

*L*-Ascorbic acid, EGCG, oleanolic acid, Trolox, DPPH, ABTS, 2,4,6-tris(2-pyridyl)-s-triazine (TPTZ), potassium persulphate (K_2_S_2_O_8_), ferrous sulfate (FeSO_4_), linoleic acid, collagenase from *Clostridium histolyticum* (EC 3.4.24.3), elastase from porcine pancreas (EC 3.4.21.36), hyaluronidase from bovine testes (EC 3.2.1.35), lyophilized tyrosinase from mushroom (EC 1.14.18.1), synthetic peptide 2-furanacryloyl-Leu-Gly-Pro-Ala (FALGPA), N-succinyl-Ala-Ala-Ala-p-nitroaniline (AAAPVN), hyaluronic acid, *L*-3,4-dihydroxyphenylalanine (*L*-DOPA), *L*-tyrosine, N-tris(hydroxymethyl)methylglycine (tricine), bovine serum albumin (BSA), sodium chloride (NaCl), calcium chloride (CaCl_2_), sodium acetate trihydrate (CH_3_COONa·3H_2_O), ammonium thiocyanate (NH_4_SCN), ferrous chloride (FeCl_3_), disodium phosphate (Na_2_HPO_4_), monosodium phosphate (NaH_2_PO_4_), SLS, and Tris(hydroxymethyl)aminomethane (Tris base) were purchased from Sigma-Aldrich (Schnelldorf, Germany). DMSO, *n*-hexane, 95% *v*/*v* ethanol, glacial acetic acid, and hydrochloric acid (HCl) were purchased from RCI Labscan Ltd. (Bangkok, Thailand). 

### 3.3. Plant Extraction

#### 3.3.1. Concrete

The dried powder of the *H. coronarium* rhizomes, leaf sheaths, and leaves was separately macerated in *n*-hexane for 3 cycles of 24 h at an ambient temperature using an Innova 2100 platform shaker (New Brunswick Scientific Co., Inc., Edison, NJ, USA) set at 150 rpm. Regarding each cycle of the maceration, the filtrate was separated via filtration through Whatman No. 1 filter paper (Danaher Corp., Washington, DC, USA) using a Bucher funnel and a suction flask attached to a vacuum pump (Anest Iwata Sparmax Co., Taipei, Taiwan). The plant residue was subsequently macerated and repeated for a total of three cycles. The filtrate from three cycles of maceration was pooled together, and *n*-hexane was removed under vacuum using a rotary evaporator (Heidolph Instruments GmbH and Co.KG, Schwabach, Germany) until dryness. The concrete from the *H. coronarium* rhizomes, leaf sheaths, and leaves was stored in a light-protected container and kept at 4 °C until usage. The yields of each concrete were calculated by using the following equation:

Yield of concrete (% *w*/*w*) = (*a*/*b*) × 100, (1)

where *a* is the weight of the concrete and *b* is the weight of the dried plant materials used in the concrete extraction.

#### 3.3.2. Absolute

The absolute was prepared from the concrete of each plant material. Each *H. coronarium* concrete was individually dissolved in 95% *v*/*v* ethanol, with the temperature set at 50 °C using a thermostatic water bath (Memmert, Schwabach, Germany). Subsequently, the waxy residue, which was not dissolved, was removed via filtration through Whatman No. 1 filter paper (Danaher Corp., Washington, DC, USA) using a Bucher funnel and a suction flask attached to a vacuum pump (Anest Iwata Sparmax Co., Taipei, Taiwan). The resulting filtrate was then cooled down to −15 °C and maintained for 24 h to allow the residual wax to solidify. Subsequently, the remaining waxy residue was removed via filtration through Whatman No. 1 filter paper (Danaher Corp., Washington, DC, USA). The resulting filtrate was subjected to a rotary evaporator (Heidolph Instruments GmbH and Co.KG, Schwabach, Germany) to remove ethanol. The absolute from the *H. coronarium* rhizomes, leaf sheaths, and leaves was stored in a light-protected container and kept at 4 °C until usage. The yields of each absolute were calculated by using the following equation:

Yield of absolute (% *w*/*w*) = (*a*/*b*) × 100, (2)

where *a* is the weight of the absolute and *b* is the weight of the dried plant materials used in the concrete extraction.

#### 3.3.3. Essential Oil

The dried powder of the *H. coronarium* leaves, leaf sheaths, or rhizomes was separately subjected to hydrodistillation using the Clevenger apparatus. The plant materials and deionized (DI) water were placed into a flask placed in a heating mantle. Upon the initiation of the evaporation, it was sustained for a duration of 2 h. Thereafter, the heating process was terminated, allowing the materials to naturally cool down to an ambient temperature. The essential oil was collected. In the case where water was collected along with the essential oil, the remaining water was removed using anhydrous sodium sulfate. The essential oil from the *H. coronarium* rhizomes, leaf sheaths, and leaves was stored in a light-protected container and kept at 4 °C until usage. The yields of each essential oil were calculated by using the following equation:

Yield of essential oil (% *w*/*w*) = (*a*/*b*) × 100, (3)

where *a* is the weight of the essential oil and *b* is the weight of the dried plant materials used in the essential oil hydrodistillation.

### 3.4. Determination of Chemical Compositions via Gas Chromatography–Mass Spectrometry (GC-MS)

The chemical compositions of each aromatic extract from *H. Coronarium* were evaluated by Thermo Scientific™ GC-TRACE 1310, MS-TSQ 9000, column TG-5SILMS 30.0 m × 0.25 mm and film thickness 0.25 μm. The oven was preheated to 70–220 °C to 3 °C/min and treated for 15 min. Injector temperature 250 °C, split flow at 20 mL/min. Helium gas was used as the carrier, set up at 1 mL/min. The headspace was incubated at 100 °C for 3 min. The mass spectrometry ion source and transfer line at 230 °C by scan start mass at 35–550 m/z. The data were acquired and subsequently analyzed using the NIST mass spectral library (Thermo Fisher Scientific, Waltham, MA, USA) as the standard reference database for mass spectral analysis.

### 3.5. Antioxidant Activities Determination

#### 3.5.1. 2,2-Diphenyl-1-picrylhydrazyl (DPPH) Radical Scavenging Assay

The capacity of the aromatic extracts from *H. coronarium* to scavenge DPPH free radicals was evaluated through a DPPH assay modified from Brem et al. (2004) and Chaiyana et al. (2017) [39,40]. Concisely, 20 μL of the sample solutions, comprising *H. coronarium* aromatic extracts dissolved in DMSO at a concentration of 1 mg/mL, was mixed with 180 μL of the DPPH solution, prepared by dissolving 167 mM DPPH in DI water. The resulting mixture was incubated for 30 min in the dark at an ambient temperature. The absorbance was then measured at 520 nm utilizing a multimode microplate reader (BMG Labtech GmbH, Ortenberg, Germany). The outcomes were expressed as the percentage of DPPH inhibition, calculated using the subsequent equation:

DPPH inhibition (%) = [(*a* − *b*)/*a*] × 100, (4)

where *a* represented the absorbance of the combination without aromatic extracts from *H. Coronarium* and *b* represented the absorbance of the combination with aromatic extracts from *H. Coronarium*. *L*-Ascorbic acid was used as the positive control. The test was performed three times.

#### 3.5.2. 2,2′-Azino-bis (3-Ethylbenzothiazoline-6-sulfonic acid) (ABTS) Assay

The capacity of the aromatic extracts from *H. coronarium* to scavenge ABTS free radicals was evaluated through an ABTS assay modified from Tachakittirungrod et al. (2007) and Chaiyana et al. (2017) [40,41]. Concisely, 20 μL of the sample solutions, comprising *H. coronarium* aromatic extracts dissolved in DMSO at a concentration of 1 mg/mL, was mixed with 180 μL of the ABTS solution, prepared by combining 7 mM ABTS with 2.45 mM potassium persulfate (K_2_S_2_O_8_) and left for 16 h in the dark, followed by a dilution with ethanol to receive an absorbance of 0.7 ± 0.1 units at 750 nm. The resulting mixture was incubated for 5 min at an ambient temperature. The absorbance was then measured at 750 nm utilizing a multimode microplate reader (BMG Labtech GmbH, Ortenberg, Germany). The outcomes were expressed as the percentage of ABTS inhibition, calculated using the subsequent equation:

ABTS inhibition (%) = [(*a − b*)/*a*] × 100, (5)

where *a* represented the absorbance of the combination without aromatic extracts from *H. Coronarium* and *b* represented the absorbance of the combination with aromatic extracts from *H. Coronarium*. *L*-Ascorbic acid was used as the positive control. The test was performed three times.

#### 3.5.3. Ferric-Reducing Antioxidant Power (FRAP) Assay

The capacity of the aromatic extracts from *H. coronarium* to reduce ferric was evaluated through an ABTS assay modified from Saeio et al. (2011) and Chaiyana et al. (2017) [40,42]. Concisely, 20 μL of the sample solutions, comprising *H. coronarium* aromatic extracts dissolved in DMSO at a concentration of 1 mg/mL, was mixed with 180 μL of the FRAP solution, prepared by combining 0.3 M acetate buffer pH 3.6, 10 mM TPTZ in 40 mM HCl solution, and 20 mM ferric chloride solution. The resulting mixture was incubated for 5 min at an ambient temperature. The absorbance was then measured at 595 nm utilizing a multimode microplate reader (BMG Labtech GmbH, Ortenberg, Germany). The outcomes were expressed as EC_1_, calculated using the subsequent equation:

EC_1_ (g FeSO_4_/g extract) = [*a* + 0.1278]/84.56, (6)

where *a* represented the absorbance of the combination with aromatic extracts from *H. Coronarium*. *L*-Ascorbic acid was used as the positive control. The test was performed three times.

### 3.6. Anti-Skin Aging Activities Determination

#### 3.6.1. Collagenase Inhibitory Activities Determination

The capacity of the aromatic extracts from *H. coronarium* to inhibit the collagenase activity was evaluated when the substrate was FALGPA through the assay modified from Thring et al. (2009) and Laothaweerungsawat et al. (2020) [36,43]. Concisely, 20 μL of the sample solutions, comprising *H. coronarium* aromatic extracts dissolved in DMSO at a concentration of 1 mg/mL, was mixed with 20 μL of 5 units/mL collagenase with the enzyme activity greater than 90% and 80 μL of Tricine buffer. The resulting mixture was incubated for 15 min at ambient temperature, and 40 μL of 2 mM FALGPA in tricine buffer pH 7.5 was added to start the reaction. Immediately, the absorbance was then kinetically measured at 340 nm for 20 min utilizing a multimode microplate reader (BMG Labtech GmbH, Ortenberg, Germany). The outcomes were expressed as the percentage of collagenase inhibition, calculated using the subsequent equation:

Collagenase inhibition (%) = [(*a − b*)/*a*] × 100, (7)

where *a* represented the absorbance of the combination without aromatic extracts from *H. Coronarium* and *b* represented the absorbance of the combination with aromatic extracts from *H. Coronarium*. EGCG was used as the positive control. The test was performed three times.

#### 3.6.2. Elastase Inhibitory Activities Determination

The capacity of the aromatic extracts from *H. coronarium* to inhibit the elastase activity was evaluated when the substrate was AAAVPN through the assay modified from Thring et al. (2009) and Laothaweerungsawat et al. (2020) [36,43]. Concisely, 10 μL of the sample solutions, comprising *H. coronarium* aromatic extracts dissolved in DMSO at a concentration of 1 mg/mL, was mixed with 40 µL of 0.03 unit/mL elastase with the enzyme activity greater than 90%. The resulting mixture was incubated for 15 min at ambient temperature, and 100 μL of 1.6 mM AAAVPN in tris HCl buffer pH 8.0 was added to start the reaction. Immediately, the absorbance was then kinetically measured at 410 nm for 20 min utilizing a multimode microplate reader (BMG Labtech GmbH, Ortenberg, Germany). The outcomes were expressed as the percentage of elastase inhibition, calculated using the subsequent equation:

Elastase inhibition (%) = [(*a − b*)/*a*] × 100, (8)

where *a* represented the absorbance of the combination without aromatic extracts from *H. Coronarium* and *b* represented the absorbance of the combination with aromatic extracts from *H. Coronarium*. Oleanolic acid was used as the positive control. The test was performed three times.

#### 3.6.3. Hyaluronidase Inhibitory Activities Determination

The capacity of the aromatic extracts from *H. coronarium* to inhibit the hyaluronidase activity was evaluated when the substrate was hyaluronic acid through the assay modified from Thring et al. (2009) and Nema et al. (2011) [36,44]. Concisely, 20 μL of the sample solutions, comprising *H. coronarium* aromatic extracts dissolved in DMSO at a concentration of 1 mg/mL, was mixed with 100 µL of 15 unit/mL hyaluronidase with the enzyme activity greater than 90%. The resulting mixture was incubated for 10 min at the temperature of 37 °C in a thermostatic incubator (BMG Labtech, Offenburg, Germany). After 100 μL of 0.03% *w*/*v* hyaluronic acid in phosphate buffer pH 5.35 was added, the mixture was incubated again for 45 min at the temperature of 37 °C. Subsequently, 1 mL of 0.1% *w*/*v* acid BSA in acetate buffer pH 3.75 was added and incubated again at an ambient temperature for 10 min. The absorbance was then measured at 600 nm utilizing a multimode microplate reader (BMG Labtech GmbH, Ortenberg, Germany). The outcomes were expressed as the percentage of hyaluronidase inhibition, calculated using the subsequent equation:

Hyaluronidase inhibition (%) = [(*a − b*)/*a*] × 100, (9)

where *a* represented the absorbance of the combination without aromatic extracts from *H. Coronarium* and *b* represented the absorbance of the combination with aromatic extracts from *H. Coronarium*. EGCG was used as the positive control. The test was performed three times.

### 3.7. Hen’s Egg–Chorioallantoic Membrane (HET-CAM) Test 

The potency of the aromatic extracts from *H. coronarium* to induce irritation was evaluated by observing atherosclerosis on the CAM using the HET-CAM test previously described by Chaiyana et al. (2017) [40], which was modified from Luepke and Kemper (1986) and Steiling et al. (1999) [45,46]. As the embryos were less than halfway through their incubation cycle, no ethical approval was required. Incubation time for hen eggs is normally 21 d, and fertilized eggs aged 7 to 9 d were used in this investigation. Concisely, the eggshell along with the inner membrane was carefully opened with forceps. Subsequently, 30 μL of the sample dissolved in 10% *v*/*v* DMSO in olive oil at a 5 mg/mL concentration was dropped onto the CAM. Within 5 min, vascular variations, including hemorrhage, lysis, and coagulation, were observed. The time when these vascular variations were first observed was recorded. Irritation score (IS) was calculated using the subsequent equation:

Irritation score (IS) = [(301 − *t*(*h*)/300) × 5] + [(301 − *t*(*l*)/300) × 7] + [(301 − *t*(*c*)/300) × 9], (10)

where *t*(*h*)*, t*(*l*), and *t*(*c*) were the time (s) when the first vascular hemorrhage, vascular lysis, and vascular coagulation occurred, respectively. IS was then categorized as follows: 0–0.9 = no irritation, 1.0–4.9 = mild irritation, 5.0–8.9 = moderate irritation, and 9.0–21.0 = severe irritation (Freire et al., 2015). An aqueous solution of (1% *w*/*v*) SLS was used as a positive control, a normal saline solution (0.9% *w*/*v* NaCl aqueous solution) was used as a negative control, and 10% *v*/*v* DMSO in olive oil was used as a vehicle control.

### 3.8. Statistical Analysis

The data were presented as the mean and standard deviation (SD). Differences among samples were analyzed by one-way ANOVA using GraphPad Prism (8.0.2, GraphPad Software, Boston, MA, USA), followed by Tukey’s post-hoc test. Differences were considered significant with *p* < 0.05.

## 4. Conclusions

Concretes, absolutes, and essential oils were successfully extracted from *H. coronarium* rhizomes, leaf sheaths, and leaves. The highest yields of the aromatic extracts were found in the concrete, followed by the absolute and essential oils. The essential oil could not be distilled from the leaf sheaths of *H. coronarium* in the present study due to the limitations of the plant materials. Nevertheless, it is worth noting that the yield was exceptionally low. The results of this study highlighted eucalyptol as the primary component in the aromatic extracts from the *H. coronarium* rhizomes and leaf sheaths, while β-caryophyllene was predominantly detected in the concrete and absolute derived from the *H. coronarium* leaves. Absolutes, especially those obtained from the rhizomes, were identified as the most powerful antioxidants. The rhizome-derived absolute demonstrated similar ABTS scavenging capabilities to *L*-ascorbic acid, with a TEAC of 1.9 ± 0.1, and the leaf-derived absolute had a TEAC of 2.2 ± 0.1 Trolox/g extract. Notably, the EC_1_ of the absolute derived from the *H. coronarium* rhizome was 0.82 ± 0.01 µg FeSO_4_/g extract, indicating its significantly higher potency compared to *L*-ascorbic acid, which had an EC_1_ of 0.43 ± 0.03 µg FeSO_4_/g extract. Moreover, the absolute extracted from the *H. coronarium* rhizomes displayed the highest level of effectiveness in inhibiting collagenase (*p* < 0.05). On the other hand, the concrete obtained from the *H. coronarium* rhizomes and leaf sheaths showed noteworthy anti-hyaluronidase activities, with inhibitions of 90.5 ± 1.6% and 87.4 ± 5.1%, respectively. Although a majority of the aromatic extracts from *H. coronarium* induced signs of irritation in the HET-CAM test, the level of irritation was not significantly different from that of the vehicle control. Hence, it is possible that the observed irritation signs may be attributed to the solvent used rather than the aromatic extracts themselves. Therefore, the absolute derived from the *H. coronarium* rhizomes would be recommended as a promising aromatic compound due to its strong antioxidant and anti-collagenase properties. This makes it a valuable candidate for further exploration as an active ingredient in cosmetic/cosmeceutical formulations for anti-aging or anti-skin wrinkles. Additional investigations on multiple doses were suggested to achieve a more thorough evaluation of their biological effects that would offer detailed insights into the effective dosage. Further cellular analysis and clinical investigations would be suggested.

## Figures and Tables

**Table 1:** Chemical compositions of aromatic extracts from *H. coronarium*.

RT (min)	Chemical Components	MW	Formula	Rhizomes			Leaf Sheaths		Leaves		
				CC	AB	EO	CC	AB	CC	AB	EO
4.01	3-Methyl-3-pentanethiol	118.2	C_6_H_14_S	0.2 ± 0.0	-	-	3.1 ± 0.5	-	0.6 ± 0.2	-	-
4.14	*α*-Thujene	136.2	C_10_H_16_	0.1 ± 0.0	-	0.2 ± 0.0	-	-	0.2 ± 0.0	-	0.2 ± 0.0
4.31	*α*-Pinene	136.2	C_10_H_16_	3.7 ± 0.0	-	7.6 ± 0.6	9.1 ± 0.1	-	6.2 ± 0.0	0.3 ± 0.0	**10.8 ± 0.2**
4.64	Camphene	136.2	C_10_H_16_	0.3 ± 0.0	-	0.8 ± 0.1	0.7 ± 0.1	-	0.2 ± 0.0	-	0.2 ± 0.0
4.82	Benzaldehyde	106.1	C_7_H_6_O	1.2 ± 0.0	-	-	**15.0 ± 3.4**	-	2.5 ± 1.1	-	-
5.05	*β*-Phellandrene	136.2	C_10_H_16_	-	-	0.1 ± 0.0	-	-	1.1 ± 0.0	-	3.6 ± 0.3
5.20	*β*-Pinene	136.2	C_10_H_16_	**12.0 ± 0.1**	1.2 ± 0.1	**19.8 ± 0.6**	**20.1 ± 1.3**	-	**21.3 ± 0.7**	0.9 ± 0.1	**31.4 ± 0.0**
5.34	*β*-Myrcene	136.2	C_10_H_16_	0.3 ± 0.0	-	0.3 ± 0.1	-	-	-	-	0.6 ± 0.0
5.81	*α*-Phellandrene	136.2	C_10_H_16_	0.2 ± 0.0	0.3 ± 0.0	0.6 ± 0.1	-	-	0.3 ± 0.1	-	0.1 ± 0.0
6.07	Terpinene	136.2	C_10_H_17_	0.2 ± 0.0	0.7 ± 0.1	0.5 ± 0.1	0.8 ± 0.0	2.2 ± 0.4	0.5 ± 0.0	0.3 ± 0.0	0.4 ± 0.0
6.39	*D*-Limonene	136.2	C_10_H_16_	1.0 ± 0.0	1.4 ± 0.1	-	5.2 ± 0.1	0.1 ± 0.1	1.2 ± 0.1	0.2 ± 0.0	5.9 ± 0.0
6.49	Eucalyptol	154.3	C_10_H_18_O	**67.8 ± 1.0**	**72.9 ± 1.9**	**53.6 ± 0.5**	**30.1 ± 4.4**	**14.0 ± 1.2**	**19.6 ± 6.0**	1.0 ± 0.2	**18.4 ± 0.4**
8.37	Linalool	154.3	C_10_H_18_O	0.7 ± 0.0	0.5 ± 0.0	0.8 ± 0.1	-	-	0.1 ± 0.1	-	0.1 ± 0.0
10.91	Borneol	154.3	C_10_H_18_O	0.5 ± 0.0	0.8 ± 0.2	0.6 ± 0.1	1.2 ± 0.0	5.4 ± 0.1	0.4 ± 0.0	-	0.3 ± 0.0
11.22	Terpinen-4-ol	154.3	C_10_H_18_O	2.5 ± 0.1	3.5 ± 0.2	3.5 ± 0.4	0.7 ± 0.0	3.7 ± 0.3	1.1 ± 0.0	0.3 ± 0.0	2.7 ± 0.1
11.74	*L*-*α*-Terpineol	154.3	C_10_H_18_O	6.2 ± 0.4	**12.5 ± 2.0**	6.2 ± 0.5	2.6 ± 0.0	**22.8 ± 0.7**	2.4 ± 0.1	0.8 ± 0.1	2.6 ± 0.1
17.67	*α*-Terpinyl acetate	196.3	C_12_H_20_O_2_	0.2 ± 0.0	0.9 ± 0.0	0.1 ± 0.0	-	3.3 ± 0.2	0.1 ± 0.1	0.2 ± 0.0	0.1 ± 0.0
20.60	*β*-Caryophyllene	204.4	C_15_H_24_	0.2 ± 0.0	1.4 ± 0.1	0.1 ± 0.0	1.1 ± 0.1	**27.2 ± 1.3**	**33.7 ± 4.4**	**84.2 ± 0.7**	9.1 ± 0.5
22.00	Humulene	205.4	C_15_H_25_	-	-	-	-	2.4 ± 0.3	1.3 ± 0.1	6.1 ± 0.1	0.5 ± 0.0
26.86	Caryophyllene oxide	220.4	C_15_H_24_O	-	-	-	-	6.8 ± 0.9	1.0 ± 0.3	3.8 ± 0.6	1.6 ± 0.2
	Total			97.3	96.2	94.7	89.6	88.0	93.7	98.0	88.7

NOTE: RT = retention time; MW = molecular weight; CC = concretes; AB = absolutes; EO = essential oils. Bolded numbers represent the major component that was detected in more than 10% of each sample.

**Table 2:** Irritation score of aromatic extracts from *H. coronarium*.

Samples		Irritation Score	Classification
Positive control (1% *w*/*v* SLS)		11.6 ± 0.0 ^a^	Severe irritation
Negative control (0.9% *w*/*v* NaCl)		0.0 ± 0.0 ^c^	No irritation
Vehicle control (10% *v*/*v* DMSO in olive oil)		4.8 ± 0.0 ^b^	Mild irritation
Rhizomes	Concrete	5.8 ± 1.4 ^b^	Moderate irritation
	Absolute	4.6 ± 0.1 ^b^	Mild irritation
	Essential oil	5.8 ± 1.4 ^b^	Moderate irritation
Leaf sheaths	Concrete	0.0 ± 0.0 ^c^	No irritation
	Absolute	4.8 ± 0.0 ^b^	Mild irritation
Leaves	Concrete	4.1 ± 0.1 ^b^	Mild irritation
	Absolute	4.7 ± 0.1 ^b^	Mild irritation
	Essential oil	7.3 ± 3.6 ^b^	Moderate irritation

NOTE: SLS = sodium lauryl sulfate; NaCl = sodium chloride; DMSO = dimethyl sulfoxide. The letters a, b, and c denote significant differences in the irritation score among the aromatic extracts from *H. coronarium* (*p* < 0.05).

## Data Availability

Data is contained within the article.

## References

[1] Pham N.K., Nguyen H.T., Nguyen Q.B. (2021). A review on the ethnomedicinal uses, phytochemistry and pharmacology of plant species belonging to *Kaempferia* L. genus (*Zingiberaceae*). Pharm. Sci. Asia..

[2] Voon K.J., Sivasothy Y., Sundralingam U., Lalmahomed A., Goh A.P. (2022). Cytotoxic Labdane Diterpenes, Norlabdane Diterpenes and Bis-Labdanic Diterpenes from the *Zingiberaceae*: A Systematic Review. J. Pharm..

[3] Rachkeeree A., Kantadoung K., Suksathan R., Puangpradab R., Page P.A., Sommano S.R. (2018). Nutritional Compositions and Phytochemical Properties of the Edible Flowers from Selected *Zingiberaceae* Found in Thailand. Front. Nutr..

[4] Ray A., Jena S., Dash B., Kar B., Halder T., Chatterjee T., Ghosh B., Panda P.C., Nayak S., Mahapatra N. (2018). Chemical diversity, antioxidant and antimicrobial activities of the essential oils from Indian populations of *Hedychium coronarium* Koen. Ind. Crops Prod..

[5] Ke Y., Abbas F., Zhou Y., Yu R., Fan Y. (2021). Auxin-responsive R2R3-MYB transcription factors HcMYB1 and HcMYB2 activate volatile biosynthesis in *Hedychium coronarium* flowers. Front. Plant Sci..

[6] Ray A., Jena S., Kar B., Sahoo A., Panda P.C., Nayak S., Mahapatra N. (2018). Volatile metabolite profiling of ten Hedychium species by gas chromatography mass spectrometry coupled to chemometrics. Ind. Crops Prod..

[7] Tavares W.R., Barreto M.D.C., Seca A.M. (2020). Uncharted source of medicinal products: The case of the *Hedychium* genus. J. Med..

[8] Tian M., Wu X., Lu T., Zhao X., Wei F., Deng G., Zhou Y. (2020). Phytochemical analysis, antioxidant, antibacterial, cytotoxic, and enzyme inhibitory activities of *Hedychium flavum* rhizome. Front. Pharmacol..

[9] Kamble K.G., Dale A.V. (2018). A review on pharmacognostic and pharmacological approach of different species of Hedychium. Indo Am. J. Pharm. Sci..

[10] Lima A.S., Junior H.N.P.C., Costa-Junior L.M., Monteiro O.S., Maia J.G.S., da Rocha C.Q. (2021). Anthelmintic effect of essential rhizome oil from *Hedychium coronarium* Koenig (*Zingiberaceae*) introduced in Northeastern Brazil. Acta Trop..

[11] da Silva C.F., Petró R.R., Almeida R.N., Cassel E., Vargas R.M. (2021). On the production and release of *Hedychium coronarium* essential oil from nanoformulations. Ind. Crops Prod..

[12] Das R., Nayak R.K. (2022). Ethnomedicinal uses, phytochemical analysis and antibacterial activity of *Hedychium coronarium* J. Koenig rhizome. Int. J. Herb. Med..

[13] Peng W., Li P., Ling R., Wang Z., Feng X., Liu J., Yan J. (2022). Diversity of volatile compounds in ten varieties of Zingiberaceae. Molecules..

[14] Alborzi S.S., Roosta A. (2022). The effect of different solvents on the production of rose concrete and rose absolute, experimental study and thermodynamic aspects using the UNIFAC model. Chem. Eng. Res. Des..

[15] Behera S., Rath S., Akhtar M.S., Naik S.K. (2018). Biotechnological intervention through tissue culture in *Hedychium coronarium*: A potential anticancer plant. Anticancer Plants: Natural Products and Biotechnological Implements: Volume 2.

[16] Sarangthem N., Talukdar N.C., Thongam B. (2013). Collection and evaluation of Hedychium species of Manipur, Northeast India. Genet. Resour. Crop Evol..

[17] Arya S., Kumar R., Prakash O., Rawat A., Mahawer S.K., Rawat D.S., de Oliveira M. (2022). *Hedychium coronarium* J. Koenig: Traditional uses, phytochemistry, biological activities and future aspects. Curr. Org. Chem..

[18] Shanmugam P.V., Yadav A., Chanotiya C.S. (2015). Enantiomer differentiation of key volatile constituents from leaves, stems, rhizome and flowers of cultivated *Hedychium coronarium* Koenig from India. J. Essent. Oil Res..

[19] Baydar H., Kineci S. (2009). Scent composition of essential oil, concrete, absolute and hydrosol from lavandin (*Lavandula x intermedia* Emeric ex Loisel. J. Essent. Oil-Bear. Plants.

[20] Parida R., Mohanty S., Nayak S. (2015). Chemical composition of essential oil from leaf and rhizome of micropropagated and conventionally grown *Hedychium coronarium* Koen. from Eastern India. J. Essent. Oil-Bear. Plants.

[21] dos Santos B.C., Barata L.E., Marques F.A., Baroni A.C., Karnos B.A., de Oliveira P.R., Guerrero P.G. (2010). Composition of leaf and rhizome essential oils of *Hedychium coronarium* Koen. from Brazil. J. Essent. Oil Res..

[22] Fu C., Li Z., Jia C., Zhang W., Zhang Y., Yi C., Xie S. (2021). Recent advances on bio-based isobutanol separation. Energy Convers. Manag. X.

[23] Tan F., Li Y., Xie Z., Bian X., Du F., Liu S., Lu P., Wang J. (2022). Geochemical Characteristics of the Jurassic Alkane Gas in the Muli Depression, South Qilian Basin: Implications for Potential of Light Oil and Condensate. Front. Environ. Sci..

[24] Abramovič H., Grobin B., Poklar Ulrih N., Cigić B. (2018). Relevance and standardization of in vitro antioxidant assays: ABTS, DPPH, and Folin–Ciocalteu. J. Chem..

[25] Benzie I.F., Strain J.J. (1996). The ferric reducing ability of plasma (FRAP) as a measure of “antioxidant power”: The FRAP assay. Anal. Biochem..

[26] Tunnisa F., Faridah D.N., Afriyanti A., Rosalina D., Syabana M.A., Darmawan N., Yuliana N.D. (2022). Antioxidant and antidiabetic compounds identification in several Indonesian underutilized *Zingiberaceae* spices using SPME-GC/MS-based volatilomics and in silico methods. Food Chem. X.

[27] Rocha Caldas G.F., Oliveira A.R.D.S., Araújo A.V., Lafayette S.S.L., Albuquerque G.S., Silva-Neto J.D.C., Costa-Silva J.H.C., Ferreira F., da Costa J.G.M., Wanderley A.G. (2015). Gastroprotective mechanisms of the monoterpene 1, 8-cineole (eucalyptol). PLoS ONE.

[28] Chrysargyris A., Mikallou M., Petropoulos S., Tzortzakis N. (2020). Profiling of essential oils components and polyphenols for their antioxidant activity of medicinal and aromatic plants grown in different environmental conditions. Agronomy.

[29] Wojtunik-Kulesza K.A., Wiśniewska R. (2022). Interactions of Selected Monoterpenes with Iron and Copper Ions Based on Ferrozine and CUPRAC Methods–the Preliminary Studies. Chem. Biodivers..

[30] Meng D., Zhang P., Zhang L., Wang H., Ho C.T., Li S., Shahidi F., Zhao H. (2017). Detection of cellular redox reactions and antioxidant activity assays. J. Funct. Foods.

[31] Aksoy L., Kolay E., Ağılönü Y., Aslan Z., Kargıoğlu M. (2013). Free radical scavenging activity, total phenolic content, total antioxidant status, and total oxidant status of endemic *Thermopsis turcica*. Saudi J. Biol. Sci..

[32] Wong Q.Y.A., Chew F.T. (2017). Defining skin aging and its risk factors: A systematic review and meta-analysis. Sci. Rep..

[33] Russell-Goldman E., Murphy G.F. (2020). The pathobiology of skin aging: New insights into an old dilemma. Am. J. Pathol..

[34] Thring T.S., Hili P., Naughton D.P. (2009). Anti-collagenase, anti-elastase and anti-oxidant activities of extracts from 21 plants. BMC Complement Altern. Med..

[35] Fattahi T., Salman S. (2019). Hyaluronic Acid Dermal Fillers. Neurotoxins Fill. Facial Esthet. Surg..

[36] Shin S., Cho S.H., Park D., Jung E. (2020). Anti-skin aging properties of protocatechuic acid in vitro and in vivo. J. Cosmet. Dermatol..

[37] Chaiyana W., Anuchapreeda S., Punyoyai C., Neimkhum W., Lee K.H., Lin W.C., Lue S.C., Viernstein H., Mueller M. (2019). *Ocimum sanctum* Linn. as a natural source of skin anti-ageing compounds. Ind. Crops Prod..

[38] Singh M., McKenzie K., Ma X. (2017). Effect of dimethyl sulfoxide on in vitro proliferation of skin fibroblast cells. J. Biotech Res..

[39] Brem B., Seger C., Pacher T., Hartl M., Hadacek F., Hofer O., Vajrodaya S., Greger H. (2004). Antioxidant dehydrotocopherols as a new chemical character of Stemona species. Phytochemistry.

[40] Oyai C., Somwongin S., Leelapornpisid P., Ingkaninan K., Waranuch N., Srivilai J., Thitipramote N., Wisuitiprot W., Schuster R. (2017). Inhibition of 5α-reductase, IL-6 secretion, and oxidation process of *Equisetum debile* Roxb. ex vaucher extract as functional food and nutraceuticals ingredients. Nutrients.

[41] Tachakittirungrod S., Okonogi S., Chowwanapoonpohn S. (2007). Study on antioxidant activity of certain plants in Thailand: Mechanism of antioxidant action of guava leaf extract. Food Chem..

[42] Saeio K., Chaiyana W., Okonogi S. (2011). Antityrosinase and antioxidant activities of essential oils of edible Thai plants. Drug Discov. Ther..

[43] Laothaweerungsawat N., Sirithunyalug J., Chaiyana W. (2020). Chemical compositions and anti-skin-ageing activities of *Origanum vulgare* L. essential oil from tropical and mediterranean region. Molecules.

[44] Nema N.K., Maity N., Sarkar B., Mukherjee P.K. (2011). Cucumis sativus fruit-potential antioxidant, anti-hyaluronidase, and anti-elastase agent. Arch. Dermatol. Res..

[45] Luepke N.P., Kemper F.H. (1986). The HET-CAM test: An alternative to the Draize eye test. Food Chem. Toxicol..

[46] Steiling W., Bracher M., Courtellemont P., De Silva O. (1999). The HET–CAM, a useful in vitro assay for assessing the eye irritation properties of cosmetic formulations and ingredients. Toxicol. In Vitro.

